# Uniparental and transgressive expression of *α-zeins* in maize endosperm of *o2* hybrid lines

**DOI:** 10.1371/journal.pone.0206993

**Published:** 2018-11-15

**Authors:** Silvana Castelli, Iride Mascheretti, Cristian Cosentino, Barbara Lazzari, Raul Pirona, Aldo Ceriotti, Angelo Viotti, Massimiliano Lauria

**Affiliations:** Istituto di Biologia e Biotecnologia Agraria, CNR, Via Alfonso Corti, Milano, Italy; Brigham Young University, UNITED STATES

## Abstract

The *α-zein* gene family encodes the most abundant storage proteins of maize (*Zea mays*) endosperm. Members of this family are expressed in a parent-of-origin manner. To characterize this phenomenon further, we investigated the expression of a subset of α-zein polypeptides in reciprocal crosses between *o2* lines that were characterized by a simplified α-zein pattern. Maize lines that suppressed the expression of α-zeins when used as female parents were identified. The suppression was cross-specific, occurring only when specific genetic backgrounds were combined. Four *α-zein* sequences that were sensitive to uniparental expression were isolated. Molecular characterization of these *α-zeins* confirmed that their expression or suppression depended on the genetic proprieties of the endosperm tissue instead of their parental origin. DNA methylation analysis of both maternally and paternally expressed *α-zeins* revealed no clear correlation between this epigenetic marker and parent-of-origin allelic expression, suggesting that an additional factor(s) is involved in this process. Genetic analyses revealed that the ability of certain lines to suppress α-zein expression was unstable after one round of heterozygosity with non-suppressing lines. Interestingly, α-zeins also showed a transgressive expression pattern because unexpressed isoforms were reactivated in both F2 and backcross plants. Collectively, our results suggest that parent-of-origin expression of specific *α-zein* alleles depends on a complex interaction between genotypes in a manner that is reminiscent of paramutation-like phenomena.

## Introduction

Parental dependency of genetic traits frequently results from unequal contributions of genetic information from male and female gametes. Examples include traits that are encoded by mitochondria and chloroplast genes, Y chromosome-linked genes, transposable elements, and biased chromosome transmission [1,2]. Alternatively, parent-of-origin effects on gene expression can result in specific gene silencing of one of the parent alleles: a phenomenon that is known as genomic imprinting [3]. In plants, genomic imprinting was originally discovered in maize, in which certain alleles of the *r1* locus, which encodes a transcription factor that is involved in seed pigmentation, are preferentially expressed upon maternal transmission [4]. Since then, several other examples of imprinted genes have been reported in maize, including specific alleles of the zein-regulator locus *dzr1* [5] and of the α-tubulins [6]. Although the functional relevance of the imprinted expression of these maize genes remains unknown, subsequent studies have demonstrated that genomic imprinting plays a key role in the normal development of the endosperm, the specialized seed tissue that supports the embryo during germination [7]. Indeed, a number of genes cause abnormal seed development in a parent-of-origin manner when they become mutated [8,9,10,11].

High-throughput genomic and post-genomic approaches, particularly RNA-Seq, have provided a more precise genome-wide survey of imprinted genes in hybrid endosperms of different plant species [12,13,14,15], including maize [16,17,18,19]. Collectively, these investigations have enabled the identification of a discrete number of genes that exhibit imprinted expression profiles. Interestingly, only a small subset of imprinted genes are conserved among plant species, and even within the same species there are examples of allelic variations in imprinting [17,20,21]. A possible explanation for this finding is that a plant’s endosperm, which is the tissue where genomic imprinting principally occurs in plants, is characterized by an epigenetic asymmetry of parental genomes: the maternal genome is hypomethylated compared to its paternal counterpart [15,17,22,23,24,25]. It has been proposed that genome-wide demethylation of the endosperm is the result of the female epigenetic reprogramming process that occurs in the central cell, the progenitor of the endosperm, and that preferentially targets transposable elements. Demethylation of these elements results in the production of small interfering RNAs that in turn are suspected to reinforce transposon silencing in the adjacent egg cell, which contributes genetic material to the next generation [22,25]. This observation has led to the idea that genes near transposable elements might become indirect targets of transposon reprogramming, resulting in imprinted expression. Imprinted genes that are conserved among species were positively selected because of their adaptive advantage to endosperm development. Differently, imprinted genes that are confined to a specific species or ecotype/variety likely reflect allelic variations of transposon-associated genes that might either lack functional relevance or lead to the production of novel seed phenotypes that are species-specific [17,20,21].

Parent-of-origin effects on gene expression have also been reported for some alleles of the *α-zeins* [26]. These genes encode the most abundant fraction of the storage protein component of the maize endosperm and form a large gene family that can be subdivided into 4 subfamilies: *z1A*, *z1B*, *z1C*, *z1D* [27].

The *z1A*, *z1B* and *z1D* genes encode the light-type proteins (zLs) with an apparent mass of 19-kDa, whereas the *z1C* genes encode the heavy-type proteins (zHs) with an apparent mass of 22 kDa.

Although α-zeins can be easily characterized by SDS-PAGE, the study of parent-of-origin effects at these loci is complicated. For example, under optimal SDS-PAGE conditions, the 22- and 19-kDa proteins can be further divided, into five size subclasses: zein heavy-type 1 and 2 (zH1 and zH2) and zein light type 1, 2 and 3 (zL1, zL2 and zL3) [28], and each subclass is composed of several polypeptides, as determined by 2D-gel analysis (IEF followed by SDS-PAGE) [28].

A previous study [29] showed that different inbred lines carrying mutant *opaque-2* (*o2*) alleles, which is the major transcription factor of 22-kDa zeins [30], have a simplified zH pattern. We took advantage of these lines to perform a more accurate investigation of the uniparental expression of these genes in maize endosperm. Our results showed that uniparental expression of α-zeins depends on a specific combination of parental genetic backgrounds. DNA methylation, an epigenetic mark that is often associated with imprinting expression [3], did not show an obvious correlation with expression, suggesting that additional factors are involved in the parent-of-origin effects that occur at these loci. Genetic analyses revealed that the ability of certain lines to suppress zHs expression is unstable after one round of heterozygosity with non-suppressing lines. The overall results support the idea that parent-of-origin expression of specific *α-zein* alleles depend on a complex interaction between genotypes in a manner that is reminiscent of paramutation-like phenomena.

## Materials and methods

### Plant material

Maize *o2* mutant lines (Table 1) were characterized and described in Ciceri et al. [29]. Plants were grown either in the greenhouse or in the field, and seeds were harvested at maturity or at 20 days after pollination (DAP). Immature seeds were frozen in liquid nitrogen and stored at -80°C.

**Table 1:** zH1 pattern of inbred lines and reciprocal crosses as determined by SDS-PAGE analysis.

Genotype		zH1 pattern
Inbred lines	NYR*o2It* (N*o2It*)	+
	W22*o2It*	+
	Bianchi*o2It*	+
	33*o2It*	+
	Rossman*o2R* (Ro*2R*)	-
	W23*o2R*	-
	Mo17*o2R* (Mo*2R*)	-
Reciprocal crosses	N*o2It* x R*o2R* (NR)	+
	R*o2R* x N*o2It* (RN)	-
	N*o2It* x W23*o2R*	+
	W23*o2R* x N*o2It*	-
	N*o2It* x M*o2R* (NM)	+
	M*o2R* x N*o2It* (MN)	+
	W22*o2It* x R*o2R*	+
	R*o2R* x W22*o2It*	-
	W22*o2It* x W23*o2R*	+
	W23*o2R* x W22*o2It*	-
	Bianchi*o2It* x R*o2R*	+
	R*o2R* x Bianchi*o2It*	+
	33*o2It* x R*o2R*	+
	R*o2R* x 33*o2It*	+
	Bianchi*o2It* x M*o2R*	+
	M*o2R* x Bianchi*o2It*	+
	Bianchi*o2It* x W23*o2R*	+
	W23*o2R* x Bianchi*o2It*	+

### Zein extraction, electrophoresis analysis and peptide sequence determination

Zeins were analyzed by SDS-PAGE and 2D-gel analysis (2D, IEF followed by SDS-PAGE) as reported in Lund et al. [26]. Specifically, 100 mg of flour was mixed with 400 μl of 70% ethanol and 2% of 2-mercaptoethanol, and the mixture was kept in agitation at room temperature overnight. Alternatively, zein extraction was conducted without 2-mercaptoethanol. By omitting this reagent, at room temperature, the protein extract was mainly composed by the α-zeins, whereas the other zein classes were less represented in the protein extract. This approach reduced the presence of 27-kDa γ zein, thus favoring detection α-zeins, especially in SDS-PAGE experiments run on a short electrophoresis apparatus. To obtain large amounts of a single polypeptide for sequence determination, pieces of gel containing each spot were extracted from a 2D gel and rerun via SDS-PAGE. Proteins were transferred from the SDS-PAGE gel to the Immobilon PVDF transfer membrane (Millipore) in transfer buffer that was composed of 20% methanol, 25 mM Tris, and 192 mM glycine at pH 8.3. Sequences at the amino-terminus were determined by automatic sequencing using an ABI-Perkin Elmer Mod. 477A (Primm).

### Nucleic acid extraction and amplification

Total DNA and RNA from 20-DAP endosperms of various genotypes were extracted by using CTAB and Trizol (ThermoFisher), respectively. RNA samples (10 μg) were treated with one unit of DNase I RNase-free (Ambion) to remove DNA contamination according to the manufacturer’s instructions. Subsequently, 2 μg of the treated RNA was used for first-strand cDNA synthesis with SuperScript II (Invitrogen) and 500 ng of oligo(dT)18 primer at 42°C for 1 h in a final volume of 25 μL. PCR was performed by using ~40 ng of genomic DNA and 5 μL of the reverse transcript (RT). For quantitative reverse transcription PCR (qRT-PCR), the cDNA reaction was diluted six-fold, and 3 μL of the diluted solution was used for the analysis using the iTaq Universal SYBR Green Super mix (Bio-Rad). The comparative CT method (ΔΔCT method) was employed for the relative quantification of gene expression, and the *GRMZM2G023418* gene was used as the endogenous reference. This gene encodes for eukaryotic translation initiation factor that is stably expressed across different maize tissues [31]. To confirm primer specificity, PCR products corresponding to each of the studied α-zeins were cloned into pGEM-T Easy vectors (Promega) and sequenced. Primers and PCR conditions are reported in S1 Table.

### Construction of *z1C* zein mini library and characterization of clones

cDNAs obtained from N*o2It* and N*o2It* x R*o2R* (NR) endosperms were used to amplify *z1C* sequences using z1CF and z1CR primers (see S1 Table) that contained *Hind*III restriction sites. The amplification products were analyzed on a 1% agarose gel and then purified using a Wizard PCR Preps kit (Promega). After a reaction with T4 DNA polymerase in the presence of 100 μM of each of the dNTPs, the fragments were cleaved with *Hind*III, treated at 65°C for 15 min, and inserted by ligation into a modified pBluescriptKS (-) vector (pBSL2A3) that was pre-digested with *Sma*I and *Hin*dIII. Plasmids were transformed into the XL1B *E*. *coli* strain (Stratagene) by electroporation. Single transformed colonies were grown overnight in LB medium, and plasmid DNA was extracted using a Wizard Minipreps DNA purification system Kit (Promega). *z1C* recombinant plasmids were characterized by digestion with the restriction enzymes *Pvu*II, *Hinc*II, *Pst*I and *Hha*I, all of which are diagnostic for their presence or absence in the coding sequences of *α-zeins*. The digested DNA was subjected to gel electrophoresis. Clones of interest were sequenced on both strands to confirm *z1C* identity.

### Methylation assays

For each sample, 500 ng of DNA was digested overnight with either a methylation-sensitive or the methylation-dependent McrBC restriction enzyme in 50 μl of reaction mix. For the MspJI methylation-dependent restriction enzyme, the digestion was incubated for 3 h in 50 μl of reaction mix. Semi-quantitative PCR (PlatinumTaq DNA Polymerase; Invitrogen) was performed with 3 μl of digested DNA (~30 ng) in a 30 μl reaction using specific primers (S1 Table). The EpiTect Bisulfite Kit (Qiagen) was used for single cytosine methylation analysis according to the manufacturer’s instructions. PCR products obtained with degenerated primers were cloned into pGEM-T Easy vectors (Promega) and sequenced. Data were analyzed with the online free software Kismeth (http://katahdin.mssm.edu/kismeth; [32]). Primers and PCR conditions are reported in S1 Table.

## Results

### Analysis of α-zein expression in reciprocal crosses of different *o2* lines

**Fig 1:**
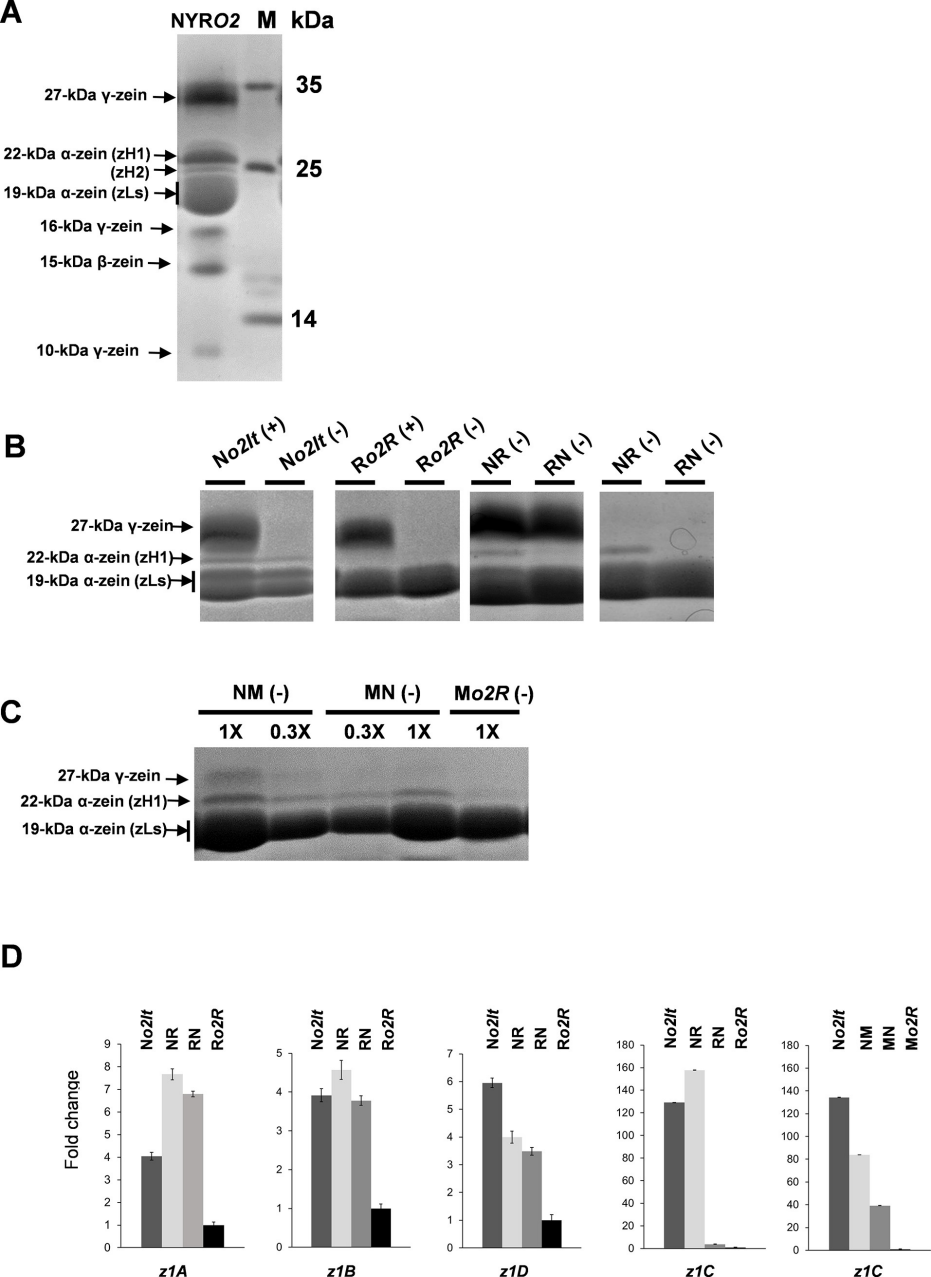
Expression analysis of α-zein in *o2* hybrid endosperms. (A) Representative example of zein pattern of a wild type endosperm (NYR) as determined by SDS-PAGE analysis. Black arrows indicate the different members of the zein family: 27-kDa, 22-kDa, 19-kDa, 16-kDa, 15-kDa, 10-kDa. (B) SDS-PAGE analysis of zein extracts of N*o2It* (zH1-plus), R*o2R* (zH1-null), N*o2It* x R*o2R* (NR) and R*o2R* x N*o2It* (RN) mature endosperms. The first genotype in the cross is the seed parent. Representative examples of zein extraction performed with (+) or without (-) 2-mercaptoethanol are shown. As discussed in the Results section, this experimental strategy permitted us to simplify the analysis of α-zeins. (C) Example of additive accumulation pattern of α-zeins between the N*o2It* and M*o2R* lines. To confirm that detection of zH1s did not depend on protein loading issues, different amount of N*o2It* x M*o2R* (NM) and M*o2R* x N*o2It* (MN) extracts were loaded. (D) Quantitative RT-PCR analysis of the four *α-zein* subfamilies (*z1A*, *z1B*, *z1C* and *z1D*) in 20-DAP endosperm. Gene expression was normalized to *GRMZM2G023418*. Data are representative of three biological replicates. Error bars represent the SD of three technical replicates.

To simplify the analysis of the parent-of-origin effects of α-zeins, we used a set of previously characterized *o2* mutant lines that exhibit simplified expression patterns of the heavy-type zeins (zHs), which can further subdivided in zH1 and zH2 types (Fig 1A). The Bianchi*o2It* (B*o2It*), NYR*o2It* (N*o2It*), W22*o2It*, and 3316*o2It* inbred lines carry the *o2Italian* (*o2It*) allele (Table 1, Fig 1B and 1C) and retain the ability to express some members of zH1s. Conversely, the Rossman*o2R* (R*o2R*), W23*o2R*, and Mo17*o2R* (M*o2R*) inbred lines carry different null-transcript *o2R* alleles and exhibit a more severe reduction of zHs (Table 1, Fig 1B and 1C) [29,33]. Accordingly, nine pairs of reciprocal crosses between the *o2It* and *o2R* lines were generated, and the expression of zH1s in hybrid endosperms was determined by SDS-PAGE. Because zein pattern accumulation is genotype specific, to facilitate the analysis, zeins were extracted mainly by omitting the 2-mercaptoethanol reducing agent. By using this approach, we improved the detection, comparison and quantification of α-zeins by reducing the presence of the other zein family members (see Materials and Methods and Fig 1B). The analysis allowed us to identify at least two pairs of reciprocal crosses in which the expression of zH1s occurred in a parent-of-origin manner (Table 1). For example, in reciprocal crosses between the N*o2It* and R*o2R* lines (NR and RN), the accumulation of zH1s preferentially occurred when the N*o2It* line was used as the female parent in the cross (Fig 1B). The uniparental expression is, however, genetic background specific, as it only occurs when specific genetic backgrounds are combined together. For example, in reciprocal crosses between the N*o2It* and M*o2R* lines, a normal zH1s accumulation was observed in both the N*o2It* x M*o2R* (NM) and M*o2R* x N*o2It* (MN) crosses (Table 1 and Fig 1C). In this study, to characterize this phenomenon further, we focused on the N*o2It* and R*o2R* lines, as these materials have been extensively analyzed over the past 20 years.

The zH1 pattern displayed by the N*o2It* and N*o2It* x R*o2R* (NR) endosperms was consistently weaker than those produced from other zein types, e.g., the light-type zeins (19-kDa). Because the endosperm is a triploid tissue that includes two maternal genomes and one paternal genome, it was important to confirm that the absence of α-zeins in the R*o2R* x N*o2It* (RN) endosperms was not the result of a dosage effect. For instance, the staining procedure had low sensitivity in detecting zH1s produced from one copy of the N*o2It* genome in RN endosperms. Therefore, the transcript levels of *α-zeins* were determined by qRT-PCR by using RNA extracted from immature endosperms (20 DAP) with specific primers for each of the four *α-zein* subfamilies, *z1A*, *z1B*, *z1C* and *z1D* [34]. Although each subfamily produced a unique transcriptional profile among N*o2It*, R*o2R* and their reciprocal hybrids, only the *z1C* subfamily produced a clear parent-of-origin expression pattern (Fig 1D). In contrast, expression analysis of *z1C* in NM and MN crosses produced an additive transcriptional pattern, which was in line with the accumulation of zH1s in mature seeds. Of note, we observed a general reduction of transcripts encoding the 19-kDa α-zeins in the R*o2R* compared to the N*o2It*. Collectively, our results suggest that i) the parent-of-origin pattern of α-zeins observed in NR and RN mature endosperms reflected the reduced amounts of their transcripts observed in the RN immature endosperms and that ii) the phenomenon is genetic background specific.

### Transcriptional analysis of *α-zein* regulators

**Fig 2:**
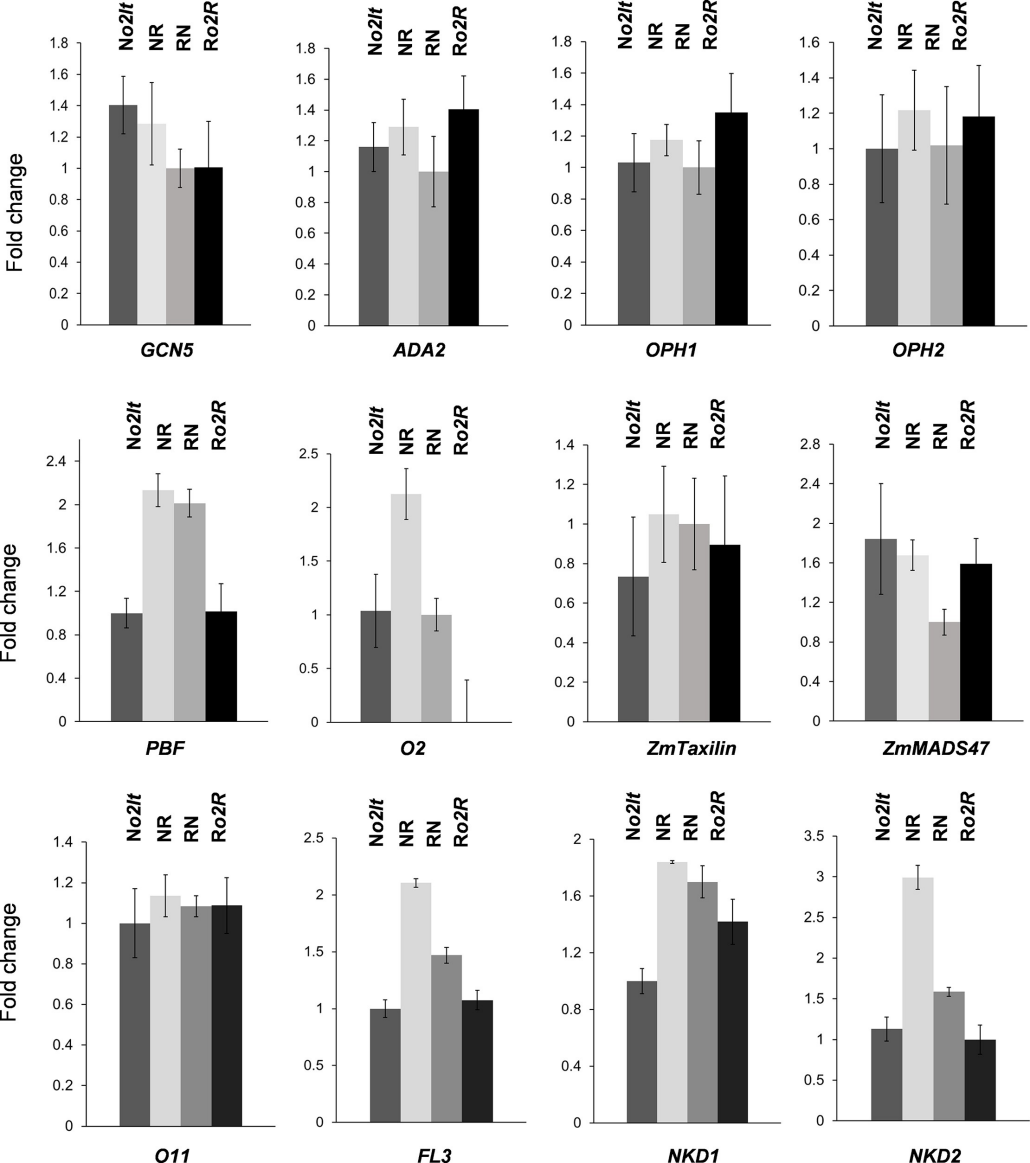
Transcriptional analysis of α-zein regulator genes. Quantitative RT-PCR analysis of *α-zein* regulator genes in 20-DAP endosperm. Gene expression was normalized to G*RMZM2G023418*. The first genotype in the cross is the seed parent. Data are representative of three biological replicates. Error bars represent the SD of three technical replicates.

The *o2It* allele encodes a protein that localizes to the nucleus and binds to the *O2* target sequence, although with a reduced efficiency compared to the wild-type protein [35]. To exclude the possibility that uniparental expression of *α-zeins* was due to imprinting regulation of their transcriptional activator, the *o2It* transcript level was analyzed by qRT-PCR in the same cDNA samples used to characterize the *α-zein* expression. In addition, the transcript levels of *OHP1/2* (*opaque-2 heterodimerizing protein 1* and *2*), *PBF* (*prolamin-box binding factor*), *GCN5* (*general control of amino-acid synthesis protein 5*), *ADA2* (*transcriptional adaptor 2*), *ZmMADS47*, and *ZmTaxilin*, *O11* (*opaque11*), *FL3* (*floury3*), *NKD1/2* (*naked endosperm 1* and *2)* were also analyzed, as these genes encode proteins that participate on *α-zein* regulation [7,36,37,38,39,40,41,42,43]. The results presented in Fig 2 show that none of the loci analyzed produced an expression pattern that could explain uniparental expression of *α-zeins* in NR and RN hybrids. It is important to note, however, that the rationale of this analysis was to detect gross transgressive transcriptional patterns between hybrids that could explain uniparental expression of *α-zeins*. Indeed, our analysis did not clarify the possible presence of regulator allelic variants between N*o2It* and R*o2R*, which may produce proteins with different proprieties, for example, weak or nonfunctional alleles that are expressed in a parent-of-origin manner.

### Identification of *α-zein* alleles that are sensitive to parent-of-origin effects

**Fig 3:**
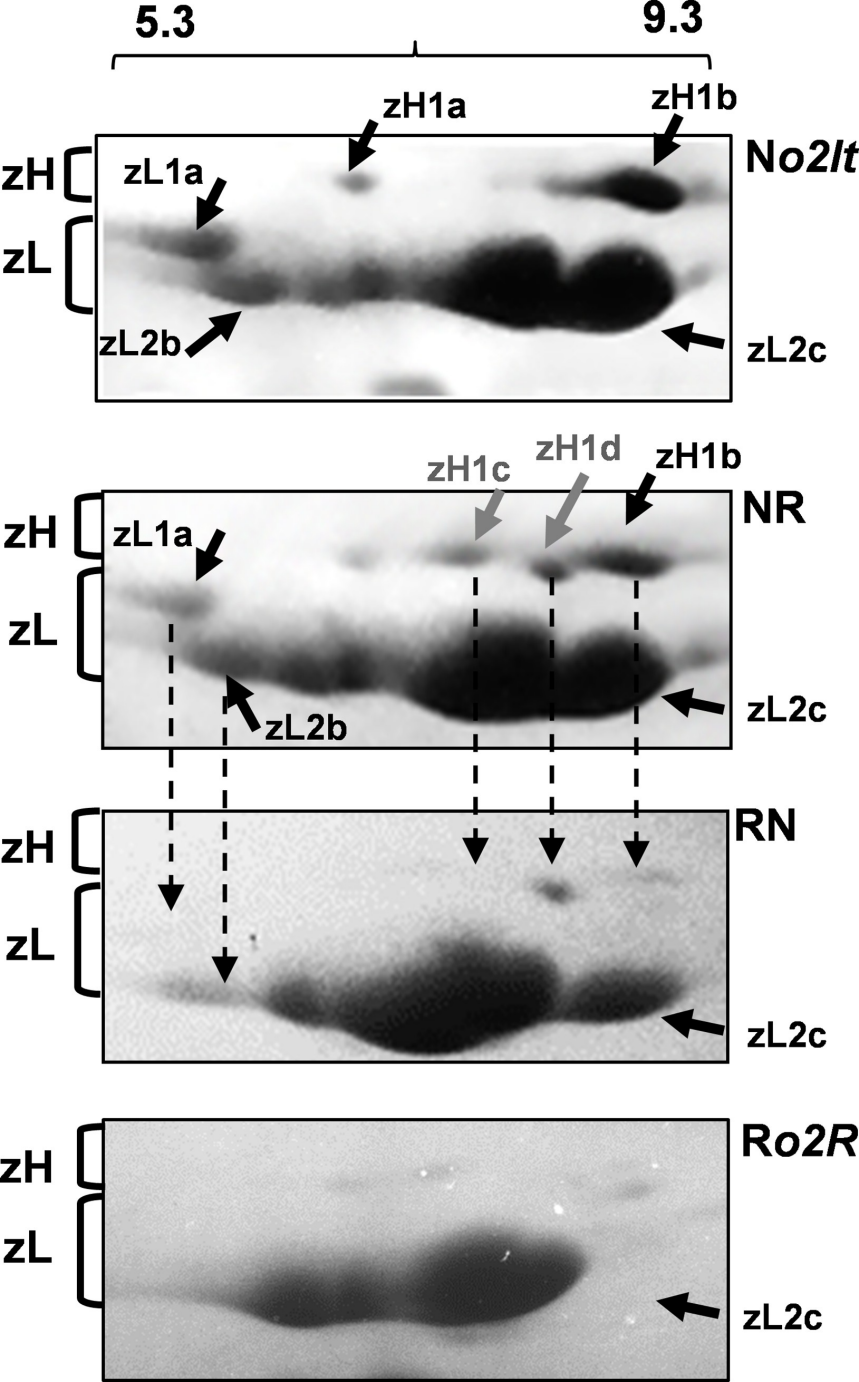
Identification of α-zein isoforms that are subject to parent-of-origin effects by 2D-gel analysis. Approximately 100 μg of zein protein extract was loaded for each genotype, with the exception of R*o2R*, for which 150 μg of zein extract was used. The numbers above the N*o2It* panel indicate the pH gradient that was used for the first dimension. The parentheses group the zH and zL isoforms. The black arrows indicate N*o2It*-specific α-zeins that were differentially detected in the reciprocal NR and RN. Gray arrows indicate R*o2R*-specific isoforms that were preferentially detected in NR endosperms. zL2c is an example of a N*o2*-specific isoform that accumulated in an additive manner.

To identify *α-zeins* that are subjected to parent-of-origin effects, we analyzed their expression in NR and RN endosperms using 2D-gel analysis. Based on the 2D profiles, three N*o2It-*specific polypeptides were differentially detectable between the two reciprocals (Fig 3; black arrows), including one heavy-type (zH1b) and two light-type (zL1a and zL2b, belonging to the group of the 19kD polypeptides). An additional N*o2It-*specific isoform (zH1a) was also differentially detectable between NR and RN endosperms. However, because of its low intensity, it remained unclear if zH1a reflected a true differentially expressed α-zein. Interestingly, 2D-gel analysis also revealed the presence of two additional isoforms, zH1c and zH1d, that were preferentially expressed in the NR endosperm (Fig 3; gray arrows). The data presented below will show that these latter isoforms belonged to the R*o2R* genotype, and they may represent those very faint zH1 spots present in the 2D-gel of R*o2R*. Although zH1d was detectable in both the reciprocals, its intensity was lower in RN, even if two maternal copies of this allele were present the endosperm. Consequently, the expression of zH1c and zH1d in NR endosperm was favored upon paternal transmission. By determining the amino-terminal sequences of zH1b, zH1d and zL1a, we found that they all matched the consensus sequence of the *z1C* subfamily (S1 Fig) [28,44]; the fact that zL1a is the product of a zH gene type is not surprising, as zH sequences with internal deletions that modify their coding capacities have been described [28,45]. To clone the full-length coding sequences of *α-zeins* subjected to parent-of-origin expression, we prepared cDNA mini-libraries of *z1C* sequences from RNAs that were extracted from N*o2It* and NR endosperms. We identified 4 clones that, based on their proprieties (length, nucleotide sequence, amino acid sequences, and *in silico*-predicted pI), were assigned to genes that encoded zL1a, zH1b, zH1c and zH1d, whose nucleotide sequences closely resembled the *azs22/D87*, *azs22/6*, *azs22/10* and *azs22/16* alleles [17,44,46], respectively (S2 Fig).

**Fig 4:**
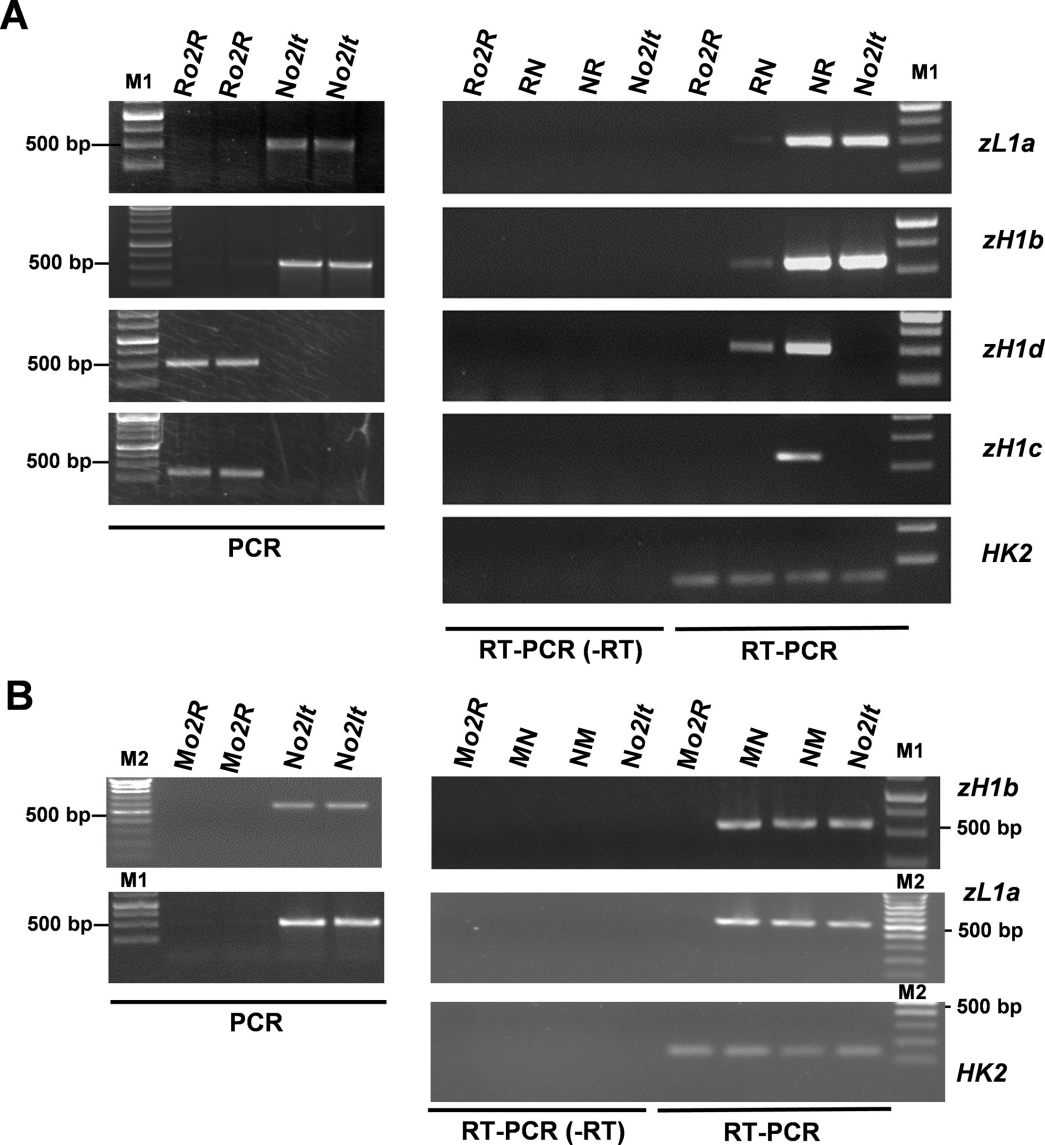
Allele specific analysis of *α-zeins*. (A) PCR and RT-PCR analysis of DNA and cDNA, respectively, of N*o2It*, NR, RN, and R*o2R* with primers specific to *zL1a*, *zH1b*, *zH1c*, *zH1d* alleles. (B) PCR and RT-PCR analysis of DNA and cDNA, respectively, of N*o2It*, NM, MN, and M*o2R* with primers specific to *zL1a*, *zH1b* alleles. For PCR analysis two independent genomic extractions were analyzed. Gene expression of G*RMZM2G023418* (*HK2*) was used as endogenous reference. RT-PCR (-RT) indicates that the reverse transcriptase enzyme was omitted in the cDNA reaction. Data are representative of two biological replicates. M1 and M2 indicate different molecular markers.

Accordingly, primers that were specific to the four sequences were designed by using the sequence information of *α-zeins* that were characterized in the B73 and BSSS53 lines [44,46]. PCR analysis performed under stringent conditions confirmed that *zH1b* and *zL1a* belonged to the N*o2It* genotype, whereas *zH1c* and *zH1b* belonged to the R*o2R* genotype (Fig 4A, PCR panel). Following this, RT-PCR analysis was performed using endosperm RNAs that were extracted from the N*o2It* and R*o2R* lines and their reciprocals. As a control, a similar analysis was performed by using RNAs extracted from M*o2R*, NM and MN endosperms. Overall, the results were in good agreement with data above. For instance, strongly maternally biased transcription was observed for *zH1b* and *zL1a*, whereas paternally biased transcription, though not exclusively, was observed for *zH1c* and *zH1b* (Fig 4A, RT-PCR panel). Finally, RT-PCR analysis confirmed that both *zL1a* and *zH1b* (N*o2It* specific) were expressed in NM and MN endosperms (Fig 4B, RT-PCR panel), supporting both the no-suppressing characteristics of the M*o2R* line on these N*o2It* alleles and the genetic background–specific nature of the phenomenon under study.

### Inheritance of *α-zein* uniparental expression in backcrosses and F2 progenies

**Fig 5:**
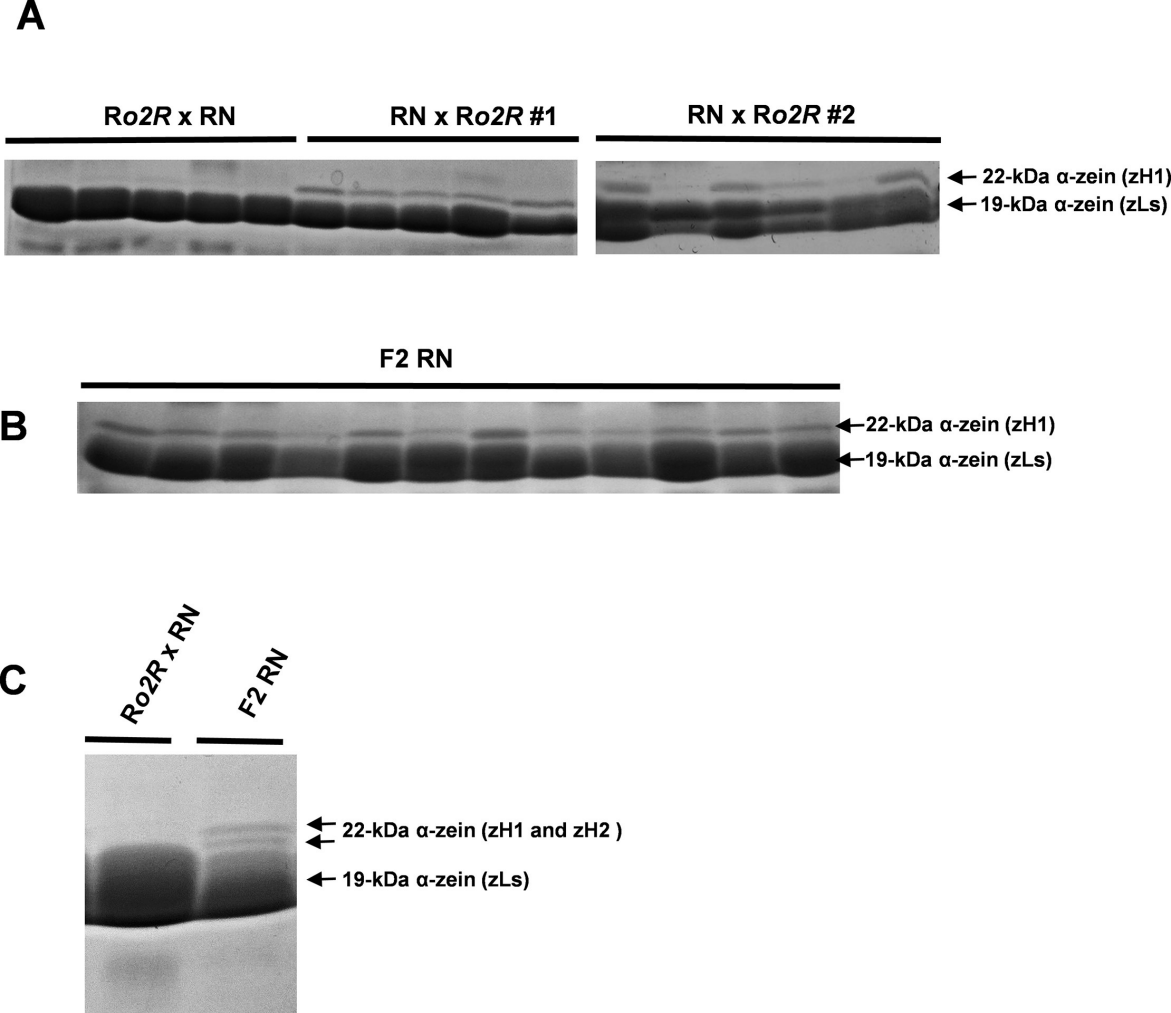
Representative examples of SDS-PAGE of α-zein extracts from backcrosses and F2 endosperm progeny. (A) Differential accumulation of the zH1 type in the reciprocal backcrosses R*o2R* x RN and RN x R*o2R*. #1 and #2 indicate that these samples come from different ears. (B) Accumulation of zH1 type in RN F2 endosperms. (C) Reactivation of zH2 type in RN F2 endosperms. The R*o2R* x RN zein extract was used as negative controls. Each lane represents a single seed zein extraction.

Expression of 10- and 18-kDa δ zeins can occur in a parent-of-origin manner due to parental imprinting of the regulatory *dzr1* locus that controls their accumulation [5,47]. Therefore, we aimed to determine whether a similar mechanism might explain the uniparental expression patterns of *α-zeins* in hybrid seeds. For clarification purposes, SDS-PAGE analysis was used to analyze F2 seeds generated from NR and RN F1 plants and seeds generated form reciprocal backcrosses between the NR and RN F1 and R*o2R* plants (Fig 5 and Table 2). If the R*o2R* and N*o2It* lines possessed a monogenic maternally expressed factor that negatively or positively regulated the accumulation of α-zeins, respectively, then the presence of zH1s should have occurred at a frequency of 50% in RN x R*o2R*, NR x R*o2R*, NR- and RN F2 seeds. In these crosses, in contrast to our expectations, we found a mixed situation. In some crosses, the expression of zH1s occurred at high frequency that ranged from 90% to 100%, whereas in other cases the expression dropped to 43% (Fig 5A and Table 2). On the other hand, R*o2R* plants that were pollinated with either RN or NR plants produced zH-null seeds with frequencies that ranged from 88% to 100% (Fig 5A and Table 2), mimicking the expression pattern observed in the RN hybrid endosperm. These results suggest that *α-zein* suppression mediated by R*o2R* can be unstable when this genotype is maternally inherited from either an RN or an NR hybrid plant. Interestingly, in some backcrosses and F2 seeds, we also observed reactivation of the zH2 zein type (Fig 5C).

**Table 2:** zH1 profile of backcross and F2 seeds as determined by SDS-PAGE analysis.

Cross	Sample	% Observed zH1 presence vs. zH1 absence	% Expected zH1 presence vs. zH1 absence ^a^
RN x N*o2It*	20	100:0	50:50
RN x R*o2R* #1	71	93:7	50:50
RN x R*o2R* #2	14	43:57	50:50
RN x R*o2R* #3	14	92:8	50:50
NR X R*o2R* #1	22	100:0	50:50
NR X R*o2R* #2	48	85:15	50:50
RN self	24	96:4	50:50
NR self	60	93:7	50:50
R*o2R* x RN	22	0:100	0:100
R*o2R* x NR #1	31	0:100	0:100
R*o2R* x NR #2	48	12:88	0:100

a; percentages are those expected if *α-zeins* expression is determined by a maternally expressed monogenic factor. The first genotype in the cross is the seed parent. When present # indicates that for a specific cross different seed progeny were analyzed.

### 3.4 DNA methylation analysis of *α-zein* alleles

**Fig 6:**
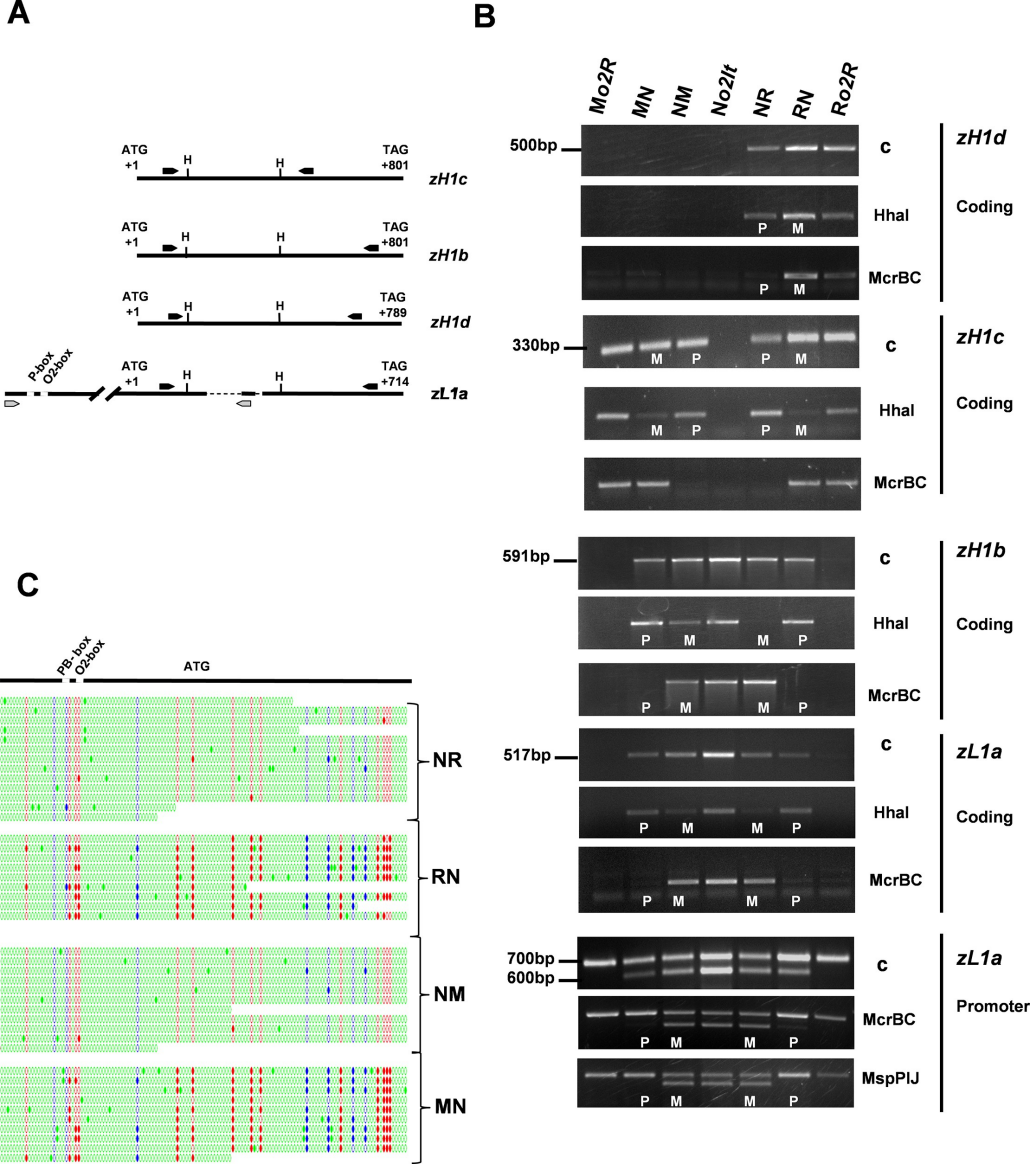
DNA methylation analysis of uniparental expressed *α-zeins*. (A) Schematic representation of the restriction maps of the *zH1b*, *zH1c*, *zH1d* and *zL1a* alleles. The positions of *Hha*I (H) restriction sites are shown. The solid bar indicates the nucleotides from the ATG to the stop codon. The dotted lines represent nucleotide deletions between *zL1a* and the other three sequences. Arrowheads indicate the positions of the primers that were used in the PCR amplification of the coding (black) and promoter regions (gray). The positions of the O2 site and the prolamin-binding site (PBS) are reported. (B) Methylation-sensitive (*Hha*I) and methylation-dependent (McrBC and MspPIJ) PCR analyses. Undigested DNA was used as a control (c). White letters indicate the parental origin of the *α-zein* alleles in the reciprocals: maternal (M), paternal (P). (C) Bisulfite sequencing analysis of the promoter and coding region of *zL1a* in the NR and RN and the NM and MN hybrid endosperms. Each line represents a single sequence. The red, blue and green colors indicate CG, CHG and CHH sites, respectively. The portion of the promoter and coding region analyzed is indicated by the graph.

Tissue-specific expression of *α-zeins* is epigenetically regulated [48,49,50,51,52]. Moreover, *α-zeins* show parental methylation differences within the endosperm [6]. Therefore, we aimed to determine whether allelic differences in DNA methylation might provide a possible explanation for the parent-of-origin effects that were observed at the studied loci. Accordingly, methylation-sensitive and methylation-dependent PCR analyses were performed using allele-specific primers to explore parental methylation differences of the *zL1a*, *zH1b*, *zH1c* and *zH1d* alleles in hybrid endosperms. Collectively, methylation-dependent PCR analysis confirmed that all of the *α-zein* alleles were subjected to maternal demethylation (Fig 6B). Conversely, methylation-sensitive PCR analysis, which detects methylation differences at specific cytosines, revealed that the extent of DNA demethylation was slightly different between alleles and hybrid genotypes (Fig 6B). The results also revealed a lack of a clear correlation between DNA methylation and zein expression. Specifically, the *zH1b* and *zL1a* alleles were paternally methylated in both the RN and MN crosses, whereas suppression was only observed in the former cross. Additionally, a single methylated paternal allele of *zH1d* produced more transcripts in NR than two demethylated maternal alleles in RN (Figs 4A and 6B). To further analyze whether a possible link existed between DNA methylation and *α-zein* expression, we analyzed the promoter region, which is more likely to affect gene expression than coding regions [53,54]. Because the *α-zein* promoters display high sequence identity and because the promoter sequences of *zH1b*, *zH1c*, *zH1d* and *zL1a* are unknown, we focused on *zL1a*. This allele closely resembles the *zp22/D87* allele (S2 Fig) and is characterized by an internal deletion that permits it to be discriminated from other paralogous sequences. Indeed, PCR analysis of the entire set of inbred lines that were used in this study revealed that only the BSSS53 (used as control) and N*o2It* line produced identical PCR patterns, which were characterized by two different PCR products (S3 Fig). The smaller product was genotype specific and identified the *zL1a;zp22/D87* allele (promoter and part of the coding region), whereas the larger product was common to all of the genotypes that were analyzed and reflected a non-specific PCR product that arose due to the high identity of zein promoter sequences. Methylation-dependent PCR analysis that was conducted with the methylation-dependent enzymes McRBC and MspJI revealed that the *zL1a* promoter region was methylated to a similar extent in both of the pairs of reciprocal crosses that were analyzed (Fig 6B). A final attempt to identify methylation differences that could explain maternal expression *vs*. paternal suppression of *α-zeins* in hybrid endosperms was made by using bisulfite sequence analysis. Specifically, we focused on a portion of both the promoter and the coding region of *zL1a*. Upon maternal transmission, *zL1a* was unmethylated in all cytosine contexts in both NR and NM hybrids. In contrast, upon paternal transmission, *zL1a* retained similar levels of CG/CHG methylation in both RN (suppressive hybrid) and MN (non-suppressive hybrid; Fig 6C).

## Discussion

The parent-of-origin effect that is exhibited by *α-zeins* in hybrid endosperms has been described as an example of genomic imprinting [26], an epigenetic mechanism that leads to monoallelic expression of genes in a parent-of-origin manner [3]. This hypothesis was supported by the observations that some *α-zein* isoforms are only maternally expressed in hybrid endosperms and that reductions in DNA methylation, an epigenetic mark that is often associated with imprinting expression, are primarily restricted to the maternal complement of *α-zeins* [26]. However, because *α-zeins* belong to a large gene family, and because of their high sequence identity, the specific sequence targets of this phenomenon are unknown. By using reciprocal crosses that were made between different *o2* genotypes and that showed a simplified *α-zein* pattern, we identified four alleles sensitive to parent-of-origin effects; this permitted us to analyze for the first time both the expression and DNA methylation patterns of these genes in a parental allele–specific manner.

The first finding of our study is that uniparental expression of *α-zeins* is not restricted to the maternal complement. For instance, in the NR endosperm, we observed *α-zeins* that exhibited both maternal- and paternal-biased gene expression. The N*o2It α-zein* alleles show an expression pattern that is consistent with maternally expressed genes, because their expression occurs in both the N*o2It* and the NR endosperms [55]. In contrast, the expression of the R*o2 α-zein* alleles was paternally enhanced in one of the two hybrid endosperms (NR), a behavior that resembled the dominance-imprinting pattern described in mice, in which the phenotype of one cross differs from the phenotype of its reciprocal and parental genotypes [55]. The coexistence of maternally and paternally expressed, or suppressed, *α-zein* alleles within the same hybrid endosperm suggests that the expression of these loci does not simply depend on their parental origin but instead on a specific mechanism/factor that is maternally provided by one of the lines used in the cross.

Indeed, even parental DNA methylation differences that we detected at these alleles did not apparently correlate with gene expression. In this regard, the best example is provided by the analysis of the N*o2It-*specific *zL1a* allele, which is maternally, expressed in both NR and NM crosses. DNA methylation profiling of its promoter region, which is considered a good predictor of transcription activity [50, 51], revealed that this allele upon maternal transmission was totally depleted of cytosine methylation in both NR and NM endosperms. On the other hand, in both RN and MN crosses *zL1a* was paternally methylated at CG and CHG sites to a similar degree in both RN and MN crosses, despite it was only suppressed in the former hybrid.

A possible explanation for this result is that our analysis did not capture differences in DNA methylation that are critical to gene expression. These differences might be present within unknown regulatory regions located either upstream or downstream of the coding region. For example, the paternal expression and maternal silencing of the Arabidopsis gene *PHERES1* depends on parental methylation differences that target repeats located 1.5–2.6 kb downstream of the 3’ end of the gene [56]. Alternatively, the silencing of *α-zeins* may require additional epigenetic marks that may or may not act in conjunction with DNA methylation. Indeed, demethylation of the 22-kDa *α-zein* promoters in maize callus does not promote *α-zein* expression even when their positive regulatory transcription factors O2 and PBF are co-expressed, suggesting that factors in addition to DNA methylation are involved in the regulation of *α-zein* expression [51].

The type of regulation observed for *α-zeins* is comparable to that already described for another zein subfamily: the *δ-zeins* [5,47]. The 10-kDa and 18-kDa δ-zeins are encoded by two genes that show parent-of-origin effects in a genetic background–specific manner as well as dominance-imprinting expression [5,47]. It has been proposed that the non-Mendelian regulation of δ-zeins is due to parental imprinting of the regulatory *dzr1* locus that post-transcriptionally controls their accumulation [5,47]. Thus, the simplest explanation is that expression of α-zeins may also depend on a regulatory factor that is itself the target of epigenetic regulation. Although the O2 protein is the main regulator of 22-kDa *α-zein*s, additional regulators have been identified [36,37,38,39,40,41,42,43], and others are likely to exist [57,58]. Interestingly, the a*zs22/D87* and a*zs22/6* alleles, namely, *zL1a* and *zH1b* in this study, respectively, are also expressed in the BSSS53*o2* line, supporting the idea that these alleles have become O2-independent [44]. Our transcriptional analysis of the *α-zein* regulators so far identified did not evidence expression patterns that may explain the uniparental expression of these genes. However, as mentioned in the Results section, our analysis only disclosed the lack of gross transgressive expression patterns at these regulatory loci in the NR and RN hybrids, and more detailed investigations will be required to know definitively whether these loci are involved in the phenomenon under study.

Supposing the existence of an endosperm factor, maternally expressed, that may either promote (N*o2It-* specific) or suppress (R*o2R*-specific) *α-zein* expression, one would expect to find the zH1-plus and zH1-null phenotype in 50% of seeds that have either the RN or the NR genotype as the maternal parent, respectively. In contrast, a penetrant zH1-null phenotype would be expected in endosperms that have the R*o2R* genotype as the maternal parent. Our results reveal a complex situation that is not readily understood. The R*o2R* x RN and R*o2R* x NR endosperms displayed a penetrant zH1-null pattern, though not exclusive, which was comparable with that observed in the RN hybrid. In contrast, a more variable expression pattern was observed in RN x R*o2R*, NR x R*o2R*, RN x RN, and NR x NR endosperms. This behavior cannot be simply explained by the presence of a monogenic factor that is provided either by the N*o2It* or R*o2R* lines within the endosperm. Even considering the eventuality that a factor is provided by the seed parent either at cytoplasmic or sporophytic level, one would expect that all seeds of the above backcrosses and F2 would express α-zeins; this is clearly not the case. The complexity of these expression patterns is further evidenced by the findings that α-zeins of the zH2 types are reactivated in some backcrosses and F2 seeds. In this case, a simple mechanism of complementation between a regulatory factor and its target loci seems unlikely, because this condition would be already satisfied in the F1 hybrids. This latter phenomenon resembles the transgressive expression patterns that is observed in tomato, where novel expression states are observed in the F2 and in advanced generation [59].

Interestingly, there is again a commonalty between the expression proprieties of α-zeins and δ-zeins. For instance, the ability of certain lines to suppress maternally the expression of the 18 kDa δ-zein is abolished after a round of heterozygosity with a non-suppressing line. To explain this finding, Wu and colleagues [47] suggested that *dzr1* might also be sensitive to paramutation: an epigenetic mechanism in which one allele causes a heritable change in the expression of a homologous allele [60]. These trans-allelic interactions are mediated by small interfering RNAs and lead to either losses or gains of DNA methylation at target loci [61,62,63]. Importantly, the same trans allelic interactions also generate transgressive expression patterns in hybrid plants and in advanced generations such as the F2 [59, 60]. We wonder if such transgressive behavior may be also depend on epigenome shock that is trigged by the epigenetic asymmetry of the endosperm parental genomes [17,64], likewise that one recently observed in the epihybrids of Arabidopsis [65].

Although at this time, our data cannot clarify the extent to which uniparental expression (F1) and transgressive expression (F2 and backcross) of α-zeins are linked, and if these phenomena might reflect, at least in part, paramutation-like phenomena, they show how gene expression strongly depends on genotype interactions that occur in hybrid/recombinant endosperms. Understanding the molecular basis of this phenomenon may help to clarify how hidden parental traits can be unlocked in advanced generations.

## Supporting information

S1 Fig Amino acid sequence comparison of α-zein identified in this study. The amino acid sequence of *zL1a*, *zH1b*, *zH1c* and *zH1d* was in silico deduced. Gaps (dotted lines) were introduced to maximize homology. At the amino-termini, the amino acids of each sequence that match those determined by the direct sequences of the two corresponding polypeptides are underlined.
(TIF)

S2 Fig Neighbor-joining tree of *α-zeins* identified in this study with *α-zeins* identified in other maize lines. Zein sequences of the BSSS53 (BSSS), B73 and W22 inbred lines [44,46,66] were aligned with the *zL1a*, *zH1b*, *zH1c* and *zH1d* sequences (black arrows), and a phylogenic Neighbor-joining tree was generated by using CLC Sequence Viewer. The light-blue boxes show the *α-zein* composition of the *z1C1* and *z1C2* loci among different inbred lines. Black numbers indicate the presence of a specific *α-zein* allele, whereas gray numbers indicate that in the N*o2It* and R*o2* lines the presence of a specific *α-zein* allele is unknown.
(TIF)

S3 Fig PCR analysis of maize lines used in this study for the presence of the *zL1a* allele. The BSSS53 and B73 inbred line are used as positive and negative control, respectively, for the presence of the *zL1a* allele.
(TIF)

S1 Table List of primers used in the study and experimental conditions used. (DOCX)
