# Supplementary figures and images for: Uniparental and transgressive expression of α-zeins in maize endosperm of o2 hybrid lines
Source: PLoS One. 2018 Nov 15;13(11):e0206993. doi: 10.1371/journal.pone.0206993 (PMC6237297; doi:10.1371/journal.pone.0206993)

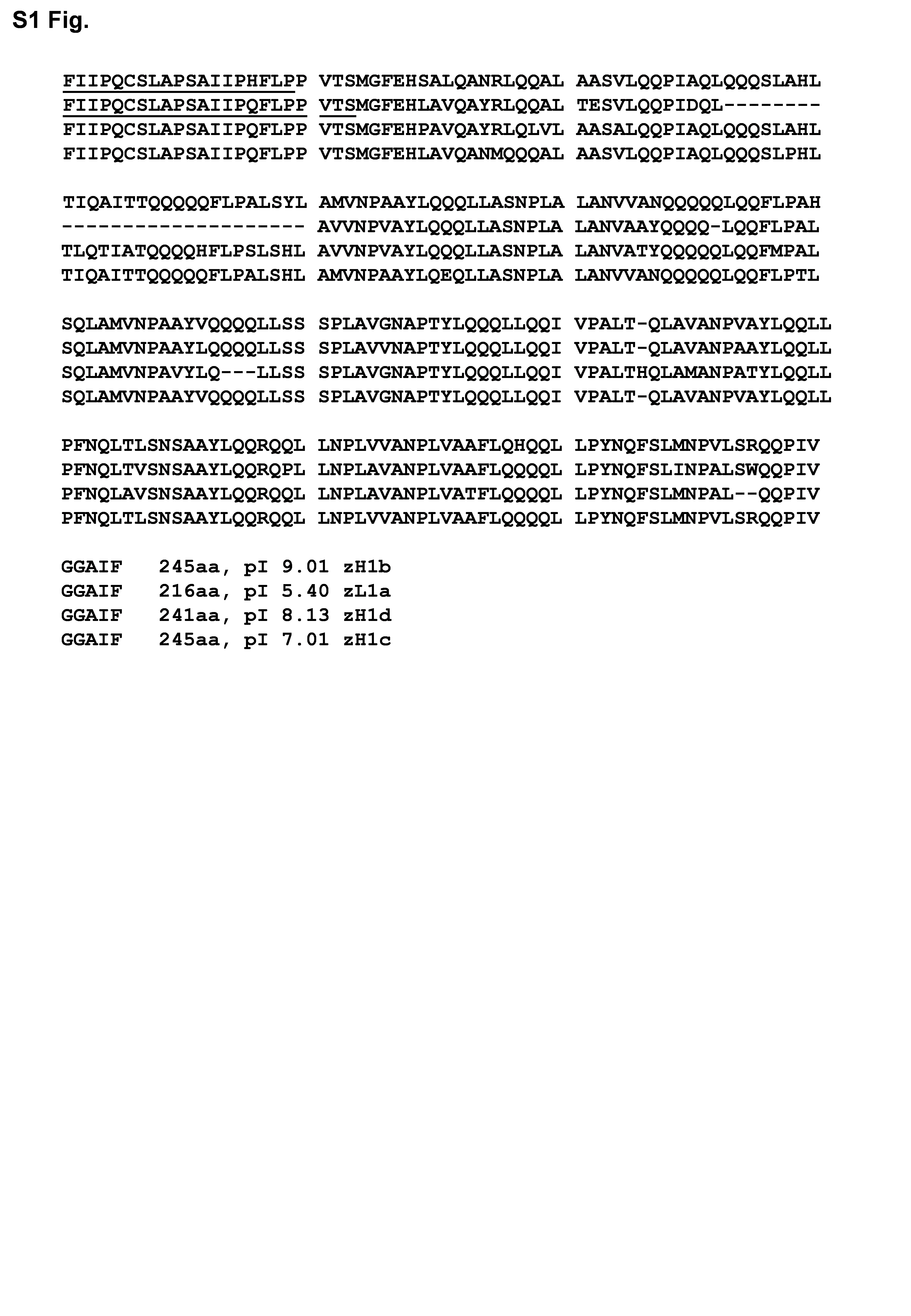

Supplement: S1 Fig — The amino acid sequence of zL1a, zH1b, zH1c and zH1d was in silico deduced. Gaps (dotted lines) were introduced to maximize homology. At the amino-termini, the amino acids of each sequence that match those determined by the direct sequences of the two corresponding polypeptides are underlined. (TIF) [file pone.0206993.s001.tif]

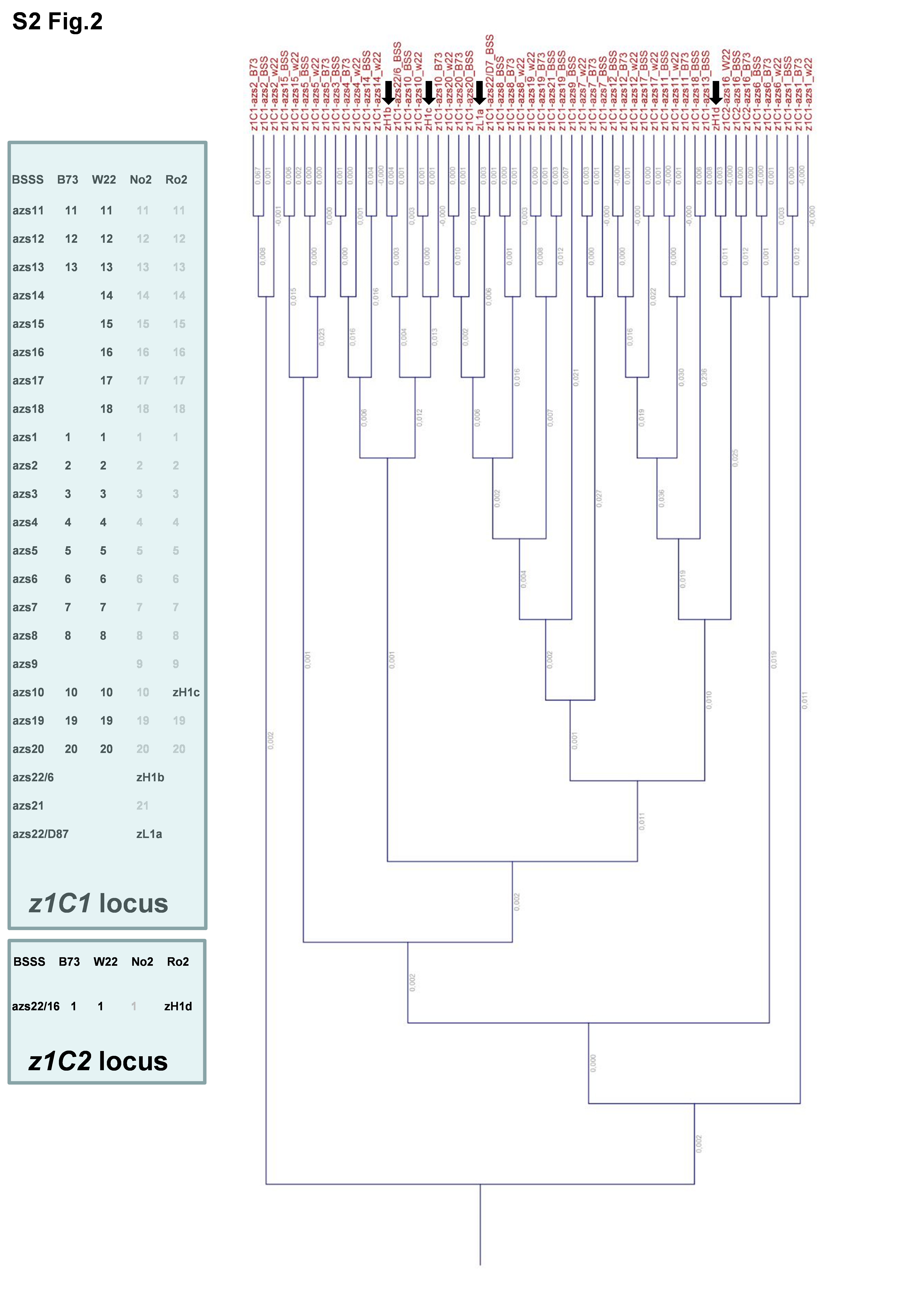

Supplement: S2 Fig — Zein sequences of the BSSS53 (BSSS), B73 and W22 inbred lines [44,46,66] were aligned with the zL1a, zH1b, zH1c and zH1d sequences (black arrows), and a phylogenic Neighbor-joining tree was generated by using CLC Sequence Viewer. The light-blue boxes show the α-zein composition of the z1C1 and z1C2 loci among different inbred lines. Black numbers indicate the presence of a specific α-zein allele, whereas gray numbers indicate that in the No2It and Ro2 lines the presence of a specific α-zein allele is unknown. (TIF) [file pone.0206993.s002.tif]

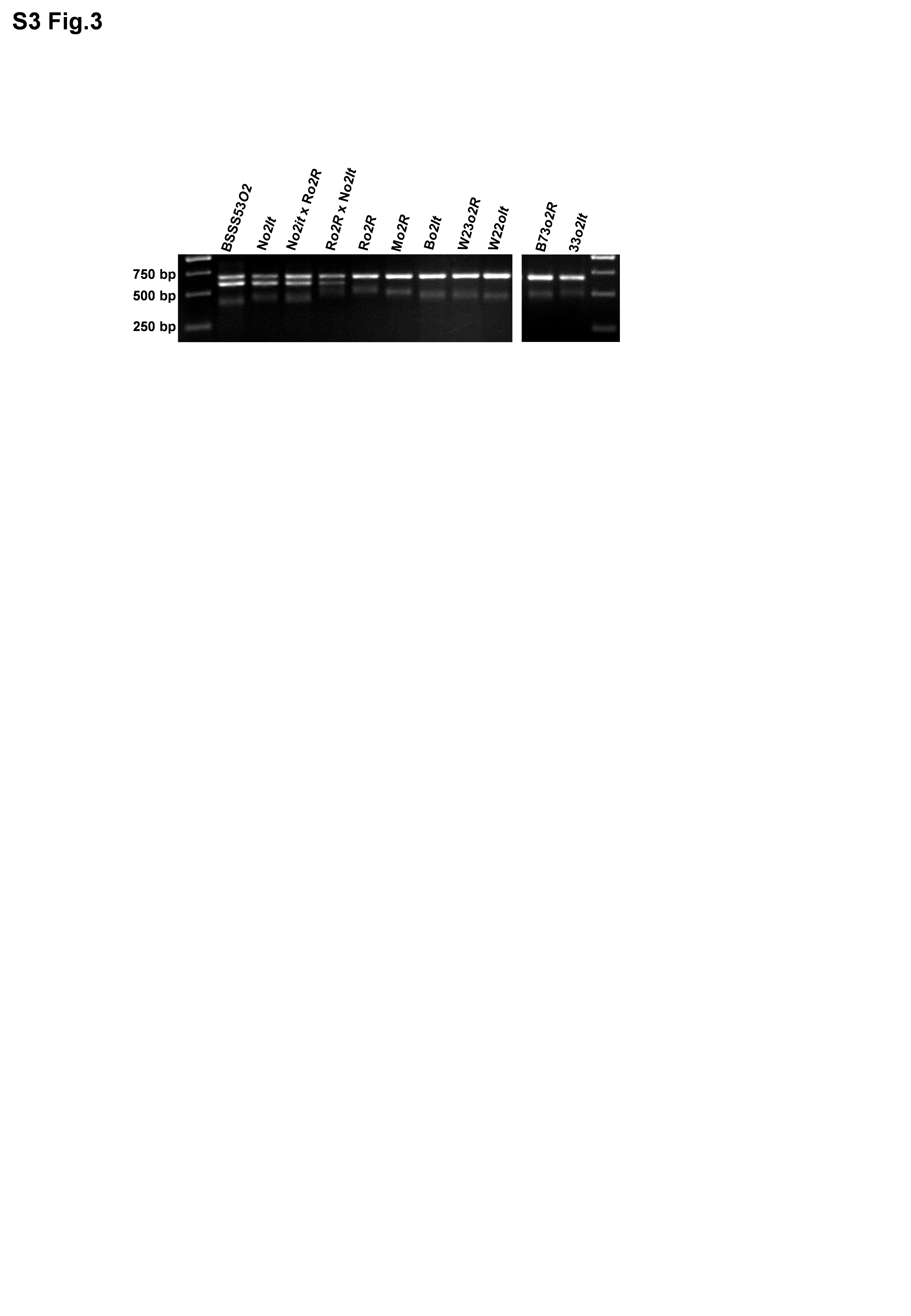

Supplement: S3 Fig — The BSSS53 and B73 inbred line are used as positive and negative control, respectively, for the presence of the zL1a allele. (TIF) [file pone.0206993.s003.tif]
