# Supplementary material for: Uniparental and transgressive expression of α-zeins in maize endosperm of o2 hybrid lines
Source: PLoS One. 2018 Nov 15;13(11):e0206993. doi: 10.1371/journal.pone.0206993 (PMC6237297; doi:10.1371/journal.pone.0206993)
Supplement: S1 Table — (DOCX) [file pone.0206993.s004.docx]

**S1 Table. List of primers used in the study and experimental conditions used.**

| Name | Sequence | Experiment | Experimental conditions |
| --- | --- | --- | --- |
| z1CF | CAATGGCTACCAAGRTATTAKCC | cDNA library | 1 cycle, 5 min, 95 °  25–35 cycles, 40 sec, 95°, 58°, and 72°  1 cycle, 5-min,72° |
| z1CR | *Hind*III  CTAT**AAGCTT**ATGTAATCTAAAAGATGGCACCTC |  |  |
| zL1aF | CTACAACAAGCGCTTACGGAG | Coding region  PCR  RT-PCR  Methyl-PCR | 1 cycle, 5 min, 95 °  31-32 cycles, 30 sec, 95°, 68°, and 72°  1 cycle, 5-min,72° |
| zL1aR | TGCCACGACAAGGCAGGGTTT |  |  |
| zH1bF | ATGGGCTTCGAACACTCAGCT | Coding region  PCR  RT-PCR  Methyl-PCR | 1 cycle, 5 min, 95 °  31-32 cycles, 30 sec, 95°, 68°, and 72°  1 cycle, 5-min,72° |
| zH1bR | CAATTGTTGATGCTGTAGGAATGC |  |  |
| zH1cF | GCTGTGCAAGCCAACATGCAAC | Coding region  PCR  RT-PCR  Methyl-PCR | 1 cycle, 5 min, 95 °  31-32 cycles, 30 sec, 95°, 68°, and 72°  1 cycle, 5-min,72° |
| zH1cR | TGAAAGCAGTTGTTGTTGTTGTAC |  |  |
| zH1dF | CAACGCAACAACAACAACATTTTC | Coding regionPCR  RT-PCR  Methyl-PCR | 1 cycle, 5 min, 95 °  31-32 cycles, 30 sec, 95°, 68°, and 72°  1 cycle, 5-min,72° |
| zH1dR | CTGCAAGGCAGGGTTCATCAAAG |  |  |
| zL1aFc | GCAAACTTTACATATCATATT | PCR promoter Methyl-PCR | 1 cycle, 5 min, 95 °  33 cycles, 30 sec, 95°, 60°, and 72°  1 cycle, 5-min,72° |
| zL1aRc | GTTCACCACAGCTAGTTGGT |  |  |
| zA1_FeF | GCTCCTTGGTCTTTCTGCAA | qRT-PCR | 1 cycle, 5 min, 95 °  40 cycles, 20 sec, 95°,  1 min 66° |
| zA1_FeR | GGTAACTGCTGTAATAGGGCTGATG |  |  |
| zB1_FeF | CCAGCCCTATCTTTGGTGCA | qRT-PCR | 1 cycle, 5 min, 95 °  40 cycles, 20 sec, 95°,  1 min 66° |
| zB1_FeR | TCAGTGCGGCCAATTGGTTA |  |  |
| zC1__FeF | TTCCACAATGCTCACTTGCT | qRT-PCR | 1 cycle, 5 min, 95 °  40 cycles, 20 sec, 95°,  1 min 66° |
| zC1_FeR | GTTGTTGTAAGACGCTCGCC |  |  |
| zC1__MaxF | CAAATGCGTTCATTATTCCACA | qRT-PCR | 1 cycle, 5 min, 95 °  40 cycles, 20 sec, 95°,  1 min 66° |
| zC1__MaxR | TGTTCGAAGCCCATTGAAGT |  |  |
| zD1_FeF | GCACAACAACTACAACAACA | qRT-PCR | 1 cycle, 5 min, 95 °  40 cycles, 20 sec, 95°,  1 min 66° |
| zD1_FeR | AATGGTAGTAGCTGTTGTGC |  |  |
| zmMADS47F | GAAGGGCAGTCGTCTGAATC | qRT-PCR | 1 cycle, 5 min, 95 °  40 cycles, 20 sec, 95°,  1 min 60° |
| zmMADS47R | CTCCCCCAATCCAGACCTAT |  |  |
| OHP1F | GATAAAGGCCCTGATGCCCACG | qRT-PCR | 1 cycle, 5 min, 95 °  40 cycles, 20 sec, 95°,  1 min 60 |
| OHP1R | CAATAGCAGTAGACCCGTAAGT |  |  |
| OHP2F | GCTGATGTTAACCAGAAGTTCAATGAA | qRT-PCR | 1 cycle, 5 min, 95 °  40 cycles, 20 sec, 95°,  1 min 60 |
| OHP2R | AGGGTACAATGCGTTCATGCCT |  |  |
| PBFF | TCAGCAGGATAACAAGGCCA | qRT-PCR | 1 cycle, 5 min, 95 °  40 cycles, 20 sec, 95°,  1 min 60 |
| PBFR | CACGGTCTGGCACACTAAC |  |  |
| GCN5F | CTAGAGATGTTTGTAGCCGACATGA | qRT-PCR | 1 cycle, 5 min, 95 °  40 cycles, 20 sec, 95°,  1 min 60 |
| GCN5R | GAGAGTTGTGCAAGCAGTACAGT |  |  |
| ADA2F | CAGGACTTACAAGTGTTAAGCAGT | qRT-PCR | 1 cycle, 5 min, 95 °  40 cycles, 20 sec, 95°,  1 min 60 |
| ADA2R | CAGGTAATGACTGGGTAGCAGTCT |  |  |
| ZmTaxilin | TCAACCTACCGTCCGTCTCAG | qRT-PCR | 1 cycle, 5 min, 95 °  40 cycles, 20 sec, 95°,  1 min 60 |
| ZmTaxilin | GCCAAGAAGTGTCAGCGACAAGT |  |  |
| O112F | CAGCAACGAGGCGTACATC | qRT-PCR | 1 cycle, 5 min, 95 °  40 cycles, 20 sec, 95°,  1 min 60 |
| O112R | GTCCTGGGTGGAGATGGTC |  |  |
| Fl32F | CATTCCACCCACCGTGTAATC | qRT-PCR | 1 cycle, 5 min, 95 °  40 cycles, 20 sec, 95°,  1 min 60 |
| Fl32R | AAGTTTGCACATCGCTGACAT |  |  |
| Nkd1F | CTGTGTGACATGGAGCACGAGGTG | qRT-PCR | 1 cycle, 5 min, 95 °  40 cycles, 20 sec, 95°,  1 min 60 |
| Nkd1R | CTTTGGTGGTCTCCTCTAGGT |  |  |
| Nkd2F | CAGCAGGAGACGAAGGTAGT | qRT-PCR | 1 cycle, 5 min, 95 °  40 cycles, 20 sec, 95°,  1 min 60 |
| Nkd2R | AGCTAAGAAGGTTGTTCTCTCATTTT |  |  |
| HK2F | CAACGTCAGAGCTGTACATTGAG | qRT-PCR | 1 cycle, 5 min, 95 °  40 cycles, 20 sec, 95°,  1 min 60 |
| HK2R | GACTGACACTACGGAAATCCAAAT |  |  |
| BSF | ATATTATGTTAAGGTTGT | Bisulfite | 1 cycle, 5 min, 95 °  38 cycles, 30 sec, 95°, 48°, and 72°  1 cycle, 5-min,72° |
| BSR | TTCACCACARCTARTTRRTCAA |  |  |
